# The effects of increased dose of exercise-based therapies to enhance motor recovery after stroke: a systematic review and meta-analysis

**DOI:** 10.1186/1741-7015-8-60

**Published:** 2010-10-13

**Authors:** Emma V Cooke, Kathryn Mares, Allan Clark, Raymond C Tallis, Valerie M Pomeroy

**Affiliations:** 1 St George's University of London, Academic Dept of Geriatric Medicine, London SW17 0RE, UK; 2 Health and Social Sciences Research Institute, Queen's Building, University of East Anglia, Norwich, NR4 7TJ, UK; 3 Health and Social Sciences Research Institute, MED Building, University of East Anglia, Norwich, Norfolk, NR4 7TJ, UK; 4 7 Valley Road, Bramhall, Cheshire, SK7 2NH, UK

## Abstract

**Background:**

Exercise-based therapy is known to enhance motor recovery after stroke but the most appropriate amount, i.e. the dose, of therapy is unknown. To determine the strength of current evidence for provision of a higher dose of the same types of exercise-based therapy to enhance motor recovery after stroke.

**Methods:**

An electronic search of: MEDLINE, EMBASE, CINHAL, AMED, and CENTRAL was undertaken. Two independent reviewers selected studies using predetermined inclusion criteria: randomised or quasi randomised controlled trials with or without blinding of assessors; adults, 18+ years, with a clinical diagnosis of stroke; experimental and control group interventions identical except for dose; exercise-based interventions investigated; and outcome measures of motor impairment, movement control or functional activity. Two reviewers independently extracted outcome and follow-up data. Effect sizes and 95% confidence intervals were interpreted with reference to risk of bias in included studies.

**Results:**

9 papers reporting 7 studies were included. Only 3 of the 7 included studies had all design elements assessed as low risk of bias. Intensity of the control intervention ranged from a mean of 9 to 28 hours over a maximum of 20 weeks. Experimental groups received between 14 and 92 hours of therapy over a maximum of 20 weeks. The included studies were heterogeneous with respect to types of therapy, outcome measures and time-points for outcome and follow-up. Consequently, most effect sizes relate to one study only. Single study effect sizes suggest a trend for better recovery with increased dose at the end of therapy but this trend was less evident at follow-up Meta-analysis was possible at outcome for: hand-grip strength, -10.1 [-19.1,-1.2] (2 studies, 97 participants); Action Research Arm Test (ARAT), 0.1 [-5.7,6.0] (3 studies, 126 participants); and comfortable walking speed, 0.3 [0.1,0.5] (2 studies, 58 participants). At follow-up, between 12 and 26 weeks after start of therapy, meta-analysis findings were: Motricity Arm, 10.7 [1.7,19.8] (2 studies, 83 participants); ARAT, 2.2 [-6.0,10.4] (2 studies, 83 participants); Rivermead Mobility, 1.0 [-0.6, 2.5] (2 studies, 83 participants); and comfortable walking speed, 0.2 [0.0,0.4] (2 studies, 60 participants).

**Conclusions:**

Current evidence provides some, but limited, support for the hypothesis that a higher dose of the same type of exercised-based therapy enhances motor recovery after stroke. Prospective dose-finding studies are required.

## Background

Exercise-based therapy is known to enhance motor recovery after stroke but the most appropriate amount, i.e. the dose, of therapy is unknown. There is strong clinical opinion that if higher doses of exercise-based therapy could be provided then motor outcome would be improved.

The possibility of a dose-response relationship between exercise-based therapy and motor recovery is supported by the findings of several systematic reviews [1-5]. However, some of the included trials in all of the published systematic reviews were not designed primarily to evaluate different doses of the same therapy. Rather, they were designed to evaluate either different types of therapy, augmentation of one therapy with another or even the effects of a therapy compared with no treatment. Consequently, the results of these systematic reviews are confounded by examination of different types as well as different intensities of therapies. Differentiation of the effects of different types and different intensities of exercise-based therapies is required.

In contrast to widely-held clinical opinion and conclusions of systematic reviews an increased dose of constraint-induced movement therapy (CIMT) given early after stroke resulted in a worse outcome than either a smaller dose of CMIT or a smaller dose of conventional therapy [6]. This unexpected finding echoes those from animal model studies which indicate that a high usage of a paretic forelimb early after experimental stroke is associated with a poorer motor outcome and an increase in size of the brain lesion [7-9] if it is provided early after stroke [10]. It is possible, therefore, that high doses of exercise-based therapy could be detrimental for motor recovery after stroke. This is not the only possibility, however, as experimental animal model studies indicate that more activity, provided in enriched environments, enhances motor recovery more than a standard housing environment [11]. In addition, preliminary investigation suggests the existence of a moderate relationship (r = 0.45, p < 0.01) between the number of repetitions of an exercise and improvement in motor function[12], post-hoc analysis of three separate research studies of the same therapy suggests greater benefit for a higher dose [13] and an exploratory study suggests benefit from higher dose of CIMT for people who were later after stroke [14] than were participants in the recent trial [6].

Whether an increased dose of exercise-based therapy is beneficial, detrimental or makes no difference to motor recovery after stroke needs to be elucidated. Well designed studies of different doses of the same therapy at different times after stroke in well characterised groups of stroke survivors are required. Before undertaking such studies a systematic review specifically investigating the effect of increased dose of exercise therapy is required to establish the current evidence-base. This paper reports a systematic review and meta-analysis designed to determine the strength of current evidence for providing a higher intensity of the same types of exercise-based therapy to enhance motor recovery after stroke.

## Methods

### Design

The design of this systematic review followed recommendations of the Cochane Collaboration. The review protocol was not published prior to this report other than as part of a PhD thesis [15].

### Search strategy

The following databases were searched electronically; US National Library of Medicine Database (MEDLINE); European Medical Database (EMBASE); Cumulative Index to Nursing and Allied Health Literature (CINHAL); Allied and Complementary Medicine Database (AMED); and Cochrane Central Register of Controlled Trials (CENTRAL). An example of the search strategy used is given in Table 1. The initial search was conducted to cover the time period from induction of the databases to November 2008 and this was updated in a subsequent search to include the period up to October 2009. The updated search (December 2008 to October 2009) did not include CINHAL because the host had changed from OVID. A decision was made not to update the CINHAL search because records identified through it in the initial search were also found in other databases.

**Table 1:** Search strategy for electronic databases


1. exp Stroke/ 2. stroke.mp. 3. cerebrovascular diseas$.mp. 4. cerebral vascular diseas$.mp. 5. cerebral vascular accident$.mp. 6. cerebrovascular accident$.mp. 7. (hemipleg$ or hemipar$).mp. 8. 6 or 4 or 1 or 3 or 7 or 2 or 5 9. exp Physical Therapy Modalities/ 10 physiotherapy.mp. 11. physical therapy.mp. 12. 11 or 10 or 9 13. randomized controlled trial.pt. 14. controlled clinical trial.pt. 15. randomised controlled trials.sh. 16. random allocation.sh. 17. double-blind method.sh. 18. single-blind method.sh. 19. 18 or 16 or 13 or 17 or 12 or 15 or 14 20. clinical trial.pt. 21. exp Clinical Trial/ 22. ((singl$ or doubl$ or treb$ or trip$) adj25 (blind$ or mask$)).ti, ab. 23. (clin$ adj25 trial$).ti, ab. 24. placebo$.ti, ab. 25. placebo.sh. 26. random$.ti, ab. 27. research design.sh. 28. 27 or 25 or 21 or 26 or 20 or 22 or 24 or 23 29. comparative study.sh. 30. exp Evaluation Studies/ 31. follow up studies.sh. 32. (contro$ or prospectiv$ or volunteer$).ti, ab. 33. 32 or 30 or 31 or 29	34. 33 or 28 or 19 35. exercis$.mp. 36. exercis$.sh. 37. exp Exercise/ 38. functional strength train$.mp. 39. activities of daily living.mp. 40. neuro facilitation.mp. 41. bobath therap$.mp. 42. motor relearn$.mp. 43. rehabilitation.mp. 44. rehabilitation.sh. 45. exp Rehabilitation/ 46. restoration of function$.mp. 47. 35 or 39 or 40 or 36 or 41 or 38 or 42 or 46 or 45 or 37 or 43 or 44 48. intensit$.mp. 49. intensit$.sh. 50. frequenc$.sh. 51. frequenc$.mp. 52. duration.mp. 53. duration.sh. 54. dose.mp. 55. dosage.mp. 56. amount.mp. 57. quantit$.mp. 58. how much.mp. 59. dos$.mp. 60. dosing.mp. = .doses.mp. 62. amounts.mp. 63. 63. 50 or 53 or 57 or 61 or 51 or 58 or 48 or 59 or 52 or 60 or 56 or 49 or 62 or 54 or 55 64. 64. 8 and 63 and 34 and 12 and 47

Abbreviations

mp = title, original title, abstract, name of substance word, subject heading word

Reference lists of all articles reporting included trials were searched for any extra possibly relevant records. If any records were identified from the hand searching of reference lists and they came from journals not included on the CENTRAL data base, the contents pages of those journals were hand searched. A hand search of our own private databases of references was also undertaken. In addition authors of included articles were contacted for any unpublished data.

### Criteria for inclusion of trials

#### Types of trial

Randomised or quasi randomised controlled trials with or without blinding of assessors;

#### Types of participants

Adults, aged over 18 years, with a clinical diagnosis of stroke

#### Types of interventions

• Experimental and control group interventions identical except for dose. Therapy dose can be described in terms of time spent in therapy and/or of effort expended [16]. Description of time includes: minutes per session; sessions per day/week; and number of days/weeks [16]. Description of effort can be made in terms of the work or power required to perform an exercise for example, resistance training and the amount of weight used [16]. For this systematic review dose refers to the total time spent in exercise-based therapy.

• Interventions investigated were exercise-based (no electrostimulation, splinting or orthotics) to facilitate muscle activity or functional ability;

#### Types of outcome measures

• Measure of motor impairment - muscle function. For example. Motricity Index, muscle tone, joint range of motion;

• Measures of motor impairment - movement control. For example. co-ordination, reaction time;

• Measure of motor activity. For example. Modified Rivermead Mobility Index, Action Research Arm Test, Functional Ambulation Categories, 9 Hole Peg Test.

### Trial selection

The identification of relevant trials was undertaken by two reviewers independently using the pre-set inclusion criteria set out on a predesigned form. Reviewers assessed the record titles and categorised each as 'definitely relevant', 'possibly relevant' or 'definitely irrelevant'. Any title that both reviewers ranked 'definitely irrelevant' was excluded. The reviewers repeated the categorisation process for abstracts and full papers in turn. They did not use the category 'possibly relevant for the full papers. Any disagreements between the reviewers were resolved through referral to full papers and discussion. Trials reported in full papers which were categorised as 'definitely irrelevant' by both reviewers were excluded and reasons documented. Trials which both reviewers categorised as 'definitely relevant' were classified as included trials for evaluation in this systematic review.

### Assessment of risk of bias

Assessment of the risk of bias in included trials was undertaken by two reviewers independently for design features using the tool developed by the Cochrane Collaboration [17]. Any disagreements between reviewers were resolved by discussion, referral to full papers and contact with authors for clarification where necessary. A risk of bias plot was produced for the review using categories of low, unclear or high risk.

### Data Extraction

Data extraction was undertaken by two reviewers independently using a predesigned form. Any disagreements were resolved by discussion and referral to the original full papers. Trial authors were contacted to clarify results when this was necessary and possible. Data was extracted on:

• Trial design, sample size and attrition;

• Participant characteristics' e.g. age, gender, site of lesion, stroke classification;

• Type of interventions;

• Dose of interventions (sum of treatment hours);

• Measures made at outcome (end of intervention period) and follow-up time-points in terms of average scores for trial groups.

### Statistical analysis of outcome and follow-up data

Analysis was undertaken, where possible, on an intention- to-treat basis. Trials were not excluded if data was unavailable for subjects who did not complete all the outcome measures. Data analysis was undertaken using the Cochrane statistical package RevMan 4.2.

Effect sizes were calculated as odds ratios (OR) and 95% confidence interval (CI) for dichotomous outcomes and as weighted mean differences (WMD and 95% CI) for continuous outcomes. WMDs were determined initially using a Fixed Effect Model. Where two or more trials had used the same outcome measure, however, and if there was evidence of heterogeneity, the WMDs were estimated from a Random Effects Model. Where it was not possible to combine and compare the outcome measures reported in different trials, then statistical results were described and tabulated individually. Sub-groups were formed by each follow-up time point. No overall analysis was done since this would involve combining subgroups based on the same individuals and could bias the results.

### Synthesis and interpretation

The results of the statistical analysis were interpreted with reference to the risk of bias in trials, and comparability of participants, types of interventions and dose of interventions.

## Results

**Figure 1:**
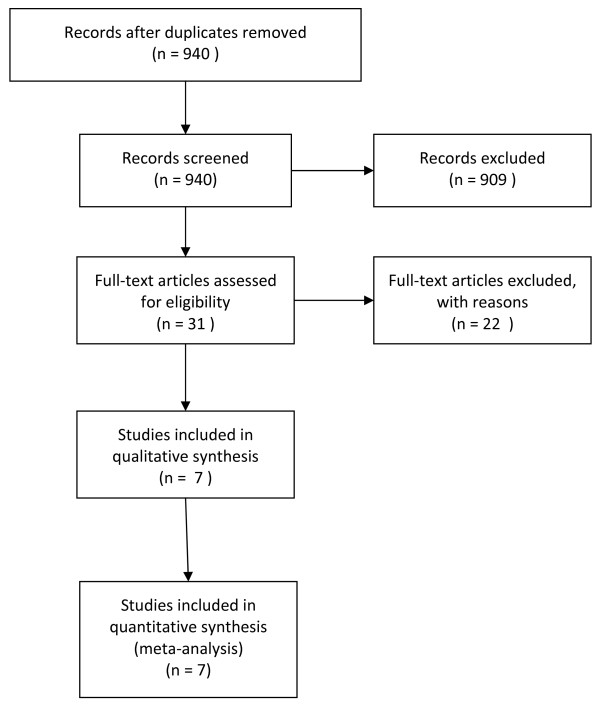
**Flow Diagram for this systematic review (note: 3 full-text articles reported the same study)**.

Full details of the number of records screened and studies included in this review are given in Figure 1. In summary, 940 potentially relevant records were screened and 31 potentially relevant records were identified. Twenty-two records did not meet the inclusion criteria and are listed in Table 2 alongside the reasons for their omission from this review. The remaining nine records were articles reporting seven studies (three articles reported different aspects of the same study [17-19]. Therefore nine articles reporting seven studies have been included in this review [18-26] (Fig 1).

**Table 2:** Excluded Studies

Study	Reason for Exclusion
Ada 2006	Not a randomised controlled trial.
Barreca 2004	Treatment interventions between control and experimental group differed in content.
Dromerick 2009	Interventions included different time periods for wearing of mitt (not an exercise based intervention) and different doses of shaping, therefore, unable to determine which aspect of this intervention would contribute to functional outcomes.
Duncan 2003	Treatment interventions between control and experimental group differed in content.
Fang 2003	Control group received no intervention, therefore study investigated effects of physiotherapy rather than an increased intensity of physiotherapy.
Feys 1998	Investigated the effects of an intervention not intensity.
Fisher 2001	Not a randomised controlled trial.
Green 2002	Investigated the effect of an intervention in a specific setting not intensity.
Kuys 2008	Not a randomised controlled trial.
Kwakkel 2002	Examination of a subgroup of the original trial (Kwakkel 1999).
Moreland 2003	Progressive resisted exercise - not the definition of intensity used in this review.
Nugent 1994	Not a controlled or randomised controlled trial.
Page 2004	Investigated the effect of an intervention not intensity.
Richards1993	Treatment interventions between control and experimental group differed in content.
Richards 2008	Not a randomised controlled trial.
Sivenius 1985	Extra therapy incorporated components of physical, occupational and speech therapy. It was not possible to isolate the effects of exercise-based therapy.
Slade 2002	Therapy analysed included physical, perceptual and cognitive, washing and dressing, daily living activities, group treatment, joint treatment and splinting and this was analysed as 'a package'. It was not possible to isolate the effects of exercise-based therapy.
Smith 1981	No specific treatment techniques described. Intensive therapy involved multi disciplinary treatment and therefore difficult to isolate the effects of exercise-based therapy. Control group also given extra treatment if deemed necessary.
Sunderland 1992	Treatment interventions between control and experimental group differed in content. The experimental group also included EMG biofeedback.
Wade 1992	Subjects received physiotherapy immediately or after three months delay, therefore effectively the first half of a crossover study - physiotherapy versus no treatment. Therefore not different intensities of the same physiotherapy treatment.
Werner 2002	Treatment interventions between control and experimental group differed in content.
Wolf 2007	Not a randomised controlled trial.

### Study designs

Of the 7 included studies three used a multi-centre, observer-blind randomised controlled design. The remaining four studies used a single-centre, observer-blind randomised controlled design (Table 3)

**Table 3:** Included studies design, participants and attrition

Study	Design	Participants										Attrition (cumulative)	
		Number & gender		Mean (SD) age (years)		Stroke lesioned hemisphere		Stroke classification		Mean (SD) time after stroke (days)			
		Control	Extra	Control	Extra	Control	Extra	Control	Extra	Control	Extra	Control	Extra
Cooke 2009	Multi-centre Observer- blind RCT	38 (21 M)	35 (22 M)	66.4 (13.7)	67.5 (11.3)	17 right	13 right	All anterior circulation stroke		36.8 (22.5)	32.4 (21.3)	7 by 6 weeks 21 by 6 weeks	3 by 6 weeks 10 by 12 weeks
Donaldson 2009	Single centre Observer- blind RCT	10 (5 M)	10 (5 M)	72.7 (14.5)	73.0 (8.6)	5 right	4 right	All anterior circulation stroke		13.4 (4.4)	25.6 (15.5)	2 by 6 weeks 7 by 12 weeks	0 by 6 weeks 4 by 12 weeks
GAPS 2004	Multi-centre Observer- blind RCT	35 (17 M)	35 (24 M)	67 (10)	68 (11)	15 right	15 right	TACI = 7 PACI = 18 LACI = 8 POCI = 1 unsure = 1	TACI = 6 PACI = 15 LACI = 10 POCI = 2 unsure = 2	25 days (range 6-71)		0 by 4 weeks 1 by 3 months 1 by 6 months	1 by 4 weeks 3 by 3 months 4 by 6 months
Lincoln 1999	Single centre Observer- blind RCT	95 (45 M)	94 (51 M)	Median 73 (IQR 64-80)	Median 73 (IQR 65-81)	38 right	47 right	TACI = 7 PACI = 29 LACI = 13 POCI = 0 unsure = 46	TACI = 9 PACI = 31 LACI = 11 POCI = 0 unsure = 43	1-5 weeks after stroke		5 by 5 weeks 11 by 3 months 14 by 6 months	7 by 5 weeks 10 by 3 months 13 by 6 months
Kwakkel 1999 & 2002	Multi-centre Observer- blind RCT	37 (14 M)	*Arm group* 33 (16 M)	64.1 (15)	*Arm group* 69 (9.8)	24 right	*Arm group* 19 right	TACI = 25 PACI = 9 LACI = 3 POCI = 0 unsure = 0	*Arm group* TACI = 19 PACI = 11 LACI = 3 POCI = 0 unsure = 0	7.5 (2.9)	*Arm group* 7.2 (2.8)	3 by 20 weeks 3 by 26 weeks 4 by 52 weeks	*Arm group* 4 by 20 weeks 4 by 26 weeks 5 by 52 weeks
		*Leg group* 21 (13 M)			*Leg group* 64.5 (9.7)		*Leg group* 18 right		*Leg group* TACI = 17 PACI = 13 LACI = 1 POCI = 0 unsure = 0		*Leg group* 7.0 (2.5)		*Leg group* 5 by 20 weeks 5 by 26 weeks 6 by 52 weeks
Partridge 2000	Single centre Observer- blind RCT	60	54	76.5 (range 60 - 90)		53 right		No data provided in paper		No data provided in paper		4 by 6 weeks 11 by 6 months	2 by 6 weeks 10 by 6 months
		(52 M)											
Rodgers 2003	Single centre Observer- blind RCT	61 (30 M)	62 (28 M)	Median 75 (no range provided)	Median 74 (no range provided)	35 right	34 right	TACI = 13 PACI = 17 LACI = 29 POCI = 2 unsure = 0	TACI = 8 PACI = 17 LACI = 34 POCI = 3 unsure = 0	Median of 5 days after stroke		10 by 3 months 13 by 6 months	8 by 3 months 14 by 6 months

### Participants

The seven studies included 680 participants (range 20-189) who completed baseline measurements (Table 3). One trial provided additional therapy from a qualified therapist and an assistant, but only the subjects treated by the qualified therapist are included in this review to ensure comparability with the other studies [22]. The mean age of participants in the seven studies ranged from 65.9 years [18-20] to 76.5 years [22] and time since stroke on admission to studies ranged from a median of 5 days [24] to a mean of 35 days [26](Table 3). Full details of participant characteristics are provided in Table 3.

### Interventions

Four of the studies described the intervention as a 'normal movement' (Bobath) approach [21-24], two studies investigated conventional physical therapy as used in the UK [25,26] and one study based the intervention on an eclectic approach involving task specific training regime following stroke [18-20] (Table 4). The dose of the exercise-based intervention was described in terms of intensity (minutes per day), frequency (times per week), duration (number of weeks). From this the total dose was calculated. The dose of the control intervention was not provided in two studies [22,23]. The dose of the control intervention in the remaining five studies ranged from a mean of 9.2 hours [26] to 27.5 hours [18-20]. The mean dose received by the experimental groups (control plus extra) ranged from 13.8 hours [25]^D ^to 91.8 hours [18-20]. Details are provided in Table 4.

**Table 4:** Included studies interventions, intensity and outcome measures

Study	Intervention		Intensity - mean hours delivered (SD)		Measurement time points				Outcome measures
	Control	Extra	Control	Extra	Baseline	Outcome	Follow-up 1	Follow-up 2	
Cooke 2009	Conventional physical therapy - lower limb from usual staff		9.2 (6.9)	23.0 (10.4)	Pre-intervention	After 6 weeks of intervention	12 weeks after end treatment	NA	▪ Walking speed ▪ Ability to walk at 0.8 m/s or more ▪ Modified Rivermead Mobility Index ▪ Knee flexion peak torque ▪ Knee extension peak torque
		Extra from research staff							
Donaldson 2009	Conventional physical therapy - upper limb from usual staff		2.81 (3.7)	13.8 (27.1)	Pre-intervention	After 6 weeks of intervention	12 weeks after end treatment		▪ Action Research Arm Test ▪ 9 hole peg test ▪ Hand grip force ▪ Pinch grip force ▪ Elbow flexion force - isometric ▪ Elbow extension force - isometric
		Extra from research staff							
GAPS 2004	Treatment broadly based on 'normal movement' (Bobath approach) from usual staff.		Average 21 (no data)	Average 34 (no data)	Pre-intervention	After 4 weeks of intervention	3 months after start treatment	6 months after start treatment	▪ Rivermead Mobility Index ▪ Motricty Index
Lincoln 1999	Treatment based on the Bobath approach from usual staff		No data	Median 9.58 extra to control (IQR 4.7-10)	Pre-intervention	After 5 weeks of intervention	3 months after start treatment	6 months after start treatment	▪ Rivermead Arm Assessment ▪ Action Research Arm Test ▪ Rivermead Motor Assess - gross function ▪ 10-hole Peg Teat ▪ Maximum grip strength
		Extra from research staff							
Kwakkel 1999 & 2002	Routine arm & leg training using evidenced-based guidelines from usual staff		27.5 arm & 23.2 leg*	*Arm group* 91.8*	Pre-intervention	After 20 weeks treatment	26 weeks after start treatment	52 weeks after start treatment	*Arm group* ▪ Action Research Arm Test ▪ Frenchay Activities Index
		*Arm group* Arm training from usual staff*Leg group* Leg training from usual staff	leg*	*Leg group* 84.2*					*Leg group* ▪ Comfortable walking speed ▪ Maximum walking speed ▪ Functional Ambulation Categories
Partridge 2000	Bobath method of treatment from usual staff		No data	No data	Pre-intervention	After 6 weeks of intervention	6 months after start treatment	NA	▪ Functional reach ▪ 5-metre timed walk ▪ Timed sit-to-stand
Rodgers 2003	Normal movement approach (Bobath) within meaningful activity and task analysis from usual staff		17.4	24.9	Pre-intervention	None	3 months after stroke	6 months after stroke	▪ Action Research Arm Test ▪ Upper Limb Motricity Index ▪ Frenchay Arm Test

* calculated using minutes/day data 20 weeks each with 5 treatment days

^$ ^calculated using median 30 days with 0.58 hours a day for control and 0.83 hours a day for extra

### Assessment of potential bias

The results of the assessment of potential bias are presented in Table 5. In summary, only 3 of the 7 included studies had all design elements assessed as low risk of bias. Of note are:

**Table 5:** Risk of bias for included studies

	Cooke 2009	Donaldson 2009	GAPS 2004	Lincoln 1999	Kwakkel 1999 & 2002	Partridge 2000	Rodgers 2000
Sequence generation	low	low	low	low	low	low	low
Allocation concealment	low	low	low	unclear	unclear	low	low
Blinding (participants, personnel and assessors)	low	low	low	low	low	low	high
Incomplete outcome data	low	low	low	unclear	low	unclear	low
Selective outcome reporting	low	low	low	low	low	high	low
Other sources of bias	low	low	low	low	low	low	low

1. The blinding procedure used in one study [24] was assessed as presenting a potential high risk of bias because in the discussion section of the paper it is stated that clinical therapists were not blind to treatment allocation and therefore gave more uni-disciplinary treatment to those participants who were receiving less therapy in the trial.

2. Allocation concealment procedures used in two studies [18-20,22] were unclear as there were no specific statements about this aspect of randomisation procedure e.g. use of sealed opaque envelopes

3. Incomplete outcome data is possibly present in two studies [22,23] but this is unclear from information presented in the papers. In one trial [22] it was reported that a number of participants died yet there was no reference to the process used for analysis for drop outs. Indeed the results tables suggest that all participants were included in the outcome analysis. One trial [23] provided no reasons for withdrawals and no methods for dealing with participants who withdrew before measurement time-points.

4. One trial appeared to report outcomes selectively [23]. Specifically step-time ratio was included in list of outcomes to be measured yet was not reported in the results section. Also sit-to-stand time, timed walk and functional reach were not measured at baseline yet there was no explanation as to why these were omitted.

### Outcomes

Extraction of data for one study [18-20] was undertaken considering its 3-group design of placebo, extra arm therapy and extra leg therapy and that all participants undertook all measures. In this present review we considered that the placebo group would act as a control for both experimental groups but that data extracted for the arm group would be that specific to the upper limb and data extracted for the leg group would be that specific to the lower limb. Consequently data analysed in this present review does not include upper limb measures reported for the leg group and vice versa. Two other studies also used a 3-group design [25,26] to compare different types and different doses of physical therapy. The data extracted from these for this review consists of that for the groups receiving the routine amount and extra amount of conventional physical therapy.

The time-points for outcome measures were mostly comparable as they were made between 4 and 6 weeks after the start of therapy except for one study where treatment was provided for 20 weeks [18-20] (Table 4) At follow-up 1 there was more variety between studies with time-points ranging from 12 to 26 weeks after the start of treatment and also 3 months after stroke (Table 4). Follow-up 2 time-points were either 6 months after start of treatment, 52 weeks after start of treatment or 6 months after stroke (Table 4)

1. Motor impairment - muscle function (Table 6)

**Table 6:** Motor impairment - muscle function

Time-point	Study	Measure used	Augmented therapy		Standard therapy		Mean difference	
			Number subjects	Mean (SD)	Number subjects	Mean (SD)	Effect size	[95% CI]
**Outcome**								
4 weeks after start therapy	GAPS	Motricity arm + leg	33	119.0 (46.0)	34	111.0 (45.0)	8.0	[-13.8,29.8]
20 weeks after start therapy	Kwakkel	Motricity leg	26	68.2 (25.8)	34	45.2 (24.8)	**23.0**	**[10.0,35.9]**
20 weeks after start therapy	Kwakkel	Motricity arm	29	53.1 (32.0)	34	28.9 (28.5)	**24.2**	**[9.2,33.1]**
6 weeks after start therapy	Donaldson	Hand grip force	10	71.9 (49.5)	8	64.8 (39.3)	7.1	[-34.0,48.1]
5 weeks after start therapy	Lincoln	Hand grip strength	87	0 (25.19)	90	11.0 (36.3)	**-11.0**	**[-20.2,-1.8]**
	***Subtotal - hand grip force/strength***		97		98		**-10.1**	**[-19.1,-1.2]**
6 weeks after start therapy	Donaldson	Pinch grip force	10	31.5 (23.1)	8	24.5 (19.7)	7.0	[-12.8,26.8]
6 weeks after start therapy	Donaldson	Elbow extend force	10	64.5 (44.6)	8	68.6 (39.6)	-4.1	[-43.1,34.8]
6 weeks after start therapy	Donaldson	Elbow flexion force	10	76.1 (58.7)	8	75.0 (38.7)	1.1	[-44.1,46.3]
6 weeks after start therapy	Cooke	Knee extend torque	26	45.3 (33.2)	25	27.8 (26.3)	**17.5^a^**	**[1.1, 33.9]**
6 weeks after start therapy	Cooke	Knee flexion torque	26	34.0 (23.1)	25	19.0 (17.8)	**15.0^a^**	**[3.7, 26.3]**
**Follow-up 1**								
3 months after start therapy	GAPS	Motricity arm + leg	32	130.0 (44.0)	33	120.0 (42.0)	10.0	[-10.9,30.9]
26 weeks after start therapy	Kwakkel	Motricity leg	26	68.2 (25.3)	34	27.2 (26.8)	**41.0**	**[27.7,54.3]**
26 weeks after start therapy	Kwakkel	Motricity arm	29	48.6 (31.1)	34	31.1 (30.1)	**17.5**	**[2.3,32.7]**
3 months after stroke	Rodgers	Motricity arm	54	85.0 (20.0)	51	78.0 (36.3)	7.0	[-4.3,18.3]
	***Subtotal - Motricity arm***		83		85		**10.7**	**[1.7,19.8]**
18 weeks after start therapy	Cooke	Knee extend torque	19	56.4 (36.3)	18	37.9 (27.8)	18.5^a^	[-2.3, 39.3]
18 weeks after start therapy	Cooke	Knee flexion torque	19	41.7 (28.8)	18	25.2 (22.9)	16.5^a^	[-0.2, 33.2]
3 months after start therapy	Lincoln	Hand grip strength	84	9.0 (28.2)	84	19.0 (43.0)	-10.0	[-19.5,1.8]
**Follow-up 2**								
6 months after start therapy	Lincoln	Hand grip strength	81	23.0 (40.7)	81	25.0 (45.2)	-2.0	[-15.3,11.3]
6 months after stroke	Rodgers	Motricity arm	48	83.0 (28.2)	48	77.0 (25.9)	6.0	[-4.8,16.8]
6 months after start therapy	GAPS	Motricity arm + leg	30	124.0 (42.0)	34	121.0 (51.0)	3.0	[-19.8,25.8]

^a ^= fixed effect model used; ^b ^= random effect model used; FU = Follow-up; * = < 0.05

Heterogeneity between studies in use of specific measures limited meta-analysis. At outcome there was a trend towards benefit for a higher dose of therapy but effect sizes for 5 of the 10 comparisons were not statistically significant. Significant effect sizes found for individual comparisons were: Motricity Index Leg score, 23.0 [10.0,35.9]; Motricity Index Arm score, 24.1 [9.2,33.1]; knee extension torque, 17.5 [1.1,33.9], knee flexion torque, 15.0 [3.7,26.3]; and hand grip strength, -11.0 [-20.2,-1.8]. Meta-analysis was only possible for hand grip force/strength (2 studies) and this found a benefit for the standard dose of therapy, -10.1 [-19.1,1.2].

At follow-up 1 the trend toward benefit for a higher dose of therapy remained but only two of the seven individual effects sizes were significant. These were both from the same study [18-20] Motricity Index Leg score, 41.0 [27.7,54.3]; and Motricity Index Arm score, 17.5 [2.3,32.7]. Meta-analysis was only possible for Motricity Index Arm score (two studies) and the effect size was 10.7 [1.7,19.8].,

No significant differences were found between the two doses of therapy at follow-up 2 (three studies). Meta-analysis was not possible.

2. Motor impairment - movement control (Table 7)

**Table 7:** Motor impairment - movement control

Time-point	Study	Measure used	Augmented therapy		Standard therapy		Mean difference	
			Number subjects	Mean (SD)	Number subjects	Mean (SD)	Effect size	[95% CI]
**Outcome**								
6 weeks after start therapy	Cooke	Symmetry step time	19	18.8 (35.6)	15	28.6 (33.1)	9.7^a^	[-32.9, 13.5]
6 weeks after start therapy	Cooke	Symmetry step length	19	13.5 (15.8)	15	25.0 (36.6)	11.5^a^	[-31.3, 8.3]
**Follow-up 1**								
18 weeks after start therapy	Cooke	Symmetry step time	19	19.4 (29.9)	14	23.0 (23.5)	3.6^a^	[-21.9, 14.6]
18 weeks after start therapy	Cooke	Symmetry step length	19	23.7 (49.9)	14	12.3 (11.0)	-11.4^a^	[-11.8, 34.6]

^a ^= fixed effect model used; ^b ^= random effect model used; FU = Follow-up; * = = < 0.05

Note: symmetry values represent difference from total symmetry therefore a higher value indicates a worse outcome.

All of the outcome measures were made at 5 or 6 weeks after the start of therapy but heterogeneity in measures used between studies prevented meta-analysis. Effect sizes were insignificant for all individual comparisons and no trends were discernable in the data.

3. Functional activity (Table 8)

**Table 8:** Effect sizes for functional activity

Time-point	Study	Measure used	Augmented therapy		Standard therapy		Mean difference	
			No. subjects	Mean (SD)	No. subjects	Mean (SD)	Effect size	[95% CI]
**Outcome**								
6 weeks after start therapy	Donaldson	ARAT	10	41.8 (17.8)	8	45.0 (14.0)	3.2	[-17.9,11.5]
20 weeks after start therapy	Kwakkel	ARAT	29	9.0 (28.9)	34	0.0 (1.5)	9.0	[-1.5,19.5]
5 weeks after start therapy	Lincoln	ARAT	87	1.0 (25.9)	90	5.0 (28.2)	-4.0	[-12.0,4.0]
	***Subtotal - ARAT***		126		132		0.1	[-5.7,6.0]
6 weeks after start therapy	Donaldson	9 Hole Peg Test	10	0.2 (0.2)	8	0.2 (0.1)	0.0^a^	[-0.1,0.1]
5 weeks after start therapy	Lincoln	10 Hole Peg Test	87	0.0 (19.3)	90	0.0 (41.5)	0.0^a^	[-9.5,9.5]
5 weeks after start therapy	Lincoln	Rivermead arm	87	3.0 (5.9)	90	4.0 (5.2)	-1.0	[-2.6,0.6]
6 weeks after start therapy	Cooke	Rivermead mobility	31	36.6 (10.4)	32	34.6 (10.8)	2.0	[-3.2,7.2]
6 weeks after start therapy	Cooke	Walk 0.8 m/s or more	31	11	32	4	**3.9^c^**	**[1.1,13.9]**
6 weeks after start therapy	Cooke	Comfort walk speed	32	0.6 (0.5)	31	0.3 (0.4)	**0.3**	**[0.1,0.5]**
20 weeks after start therapy	Kwakkel	Comfort walk speed	26	0.7 (0.5)	34	0.4 (0.4)	**0.3**	**[0.1,0.5]**
	***Subtotal - comfort walk speed***		58		65		**0.3**	**[0.1,0.5]**
20 weeks after start therapy	Kwakkel	Max walk speed	26	0.9 (0.7)	34	0.5 (0.6)	**0.4**	**[0.1,0.7]**
20 weeks after start therapy	Kwakkel	FAC	29	4 (1.5)	34	3 (2.2)	**1.0**	**[0.1,2.0]**
6 weeks after start therapy	Partridge	5 metre walk time	33	49.2 (32.0)	22	39.9 (29.9)	9.3	[-7.3,25.9]
5 weeks after start therapy	Lincoln	Rivermead Gross Function	87	3.0 (4.4)	87	5.0 (5.2)	**-2.0**	**[-3.4,-0.6]**
**Follow-up 1**								
26 weeks after start therapy	Kwakkel	ARAT	29	4.0 (28.2)	34	0.0 (1.85)	**4.0**	**[-6.3,14.3]**
3 months after stroke	Rodgers	ARAT	54	53.0 (27.4)	51	54.0 (41.5)	-1.0	[-14.5,12.5]
	***Subtotal - ARAT***		83		85		2.2	[-6.0, 10.4]
18 weeks after start therapy	Cooke	Rivermead mobility	28	36.6 (9.8)	23	39.7 (5.7)	-3.1	[-7.4,1.2]
3 months after start therapy	GAPS	Rivermead mobility	32	9.7 (3.3)	34	8.1 (3.6)	1.6	[-0.1,3.3]
	***Subtotal - Rivermead mobility***		60		57		1.0	[-0.6,2.5]
18 weeks after start therapy	Cooke	Comfort walk speed	27	0.6 (0.5)	23	0.4 (0.4)	0.2	[-0.1,0.5]
26 weeks after start therapy	Kwakkel	Comfort walk speed	26	0.6 (0.5)	34	0.4 (0.4)	0.2	[-0.0,0.4]
	***Subtotal - Comfort walk speed***		59		61		0.2	[-0.1,0.4]
3 months after stroke	Rodgers	Frenchay Arm Test	54	4.0 (2.2)	51	4.0 (3.7)	0.0	[-1.2,1.2]
3 months after start therapy	Lincoln	Rivermead arm	84	3.0 (5.9)	84	5.0 (5.2)	**-2.0**	**[-3.7,-0.3]**
6 months after start therapy	Partridge	5 metre walk time	27	35.8 (16.5)	33	49.4 (32.1)	**-13.6**	**[-26.2,-1.0]**
3 months after start therapy	Lincoln	Rivermead Gross Function	84	5.0 (5.2)	84	6.0 (5.9)	-1.0	[-2.7,0.7]
26 weeks after start therapy	Kwakkel	FAC	26	5.0 (0.7)	34	4.0 (2.2)	**1.0**	**[0.2,1.8]**
18 weeks after start therapy	Cooke	Walk 0.8 m/s or more	27	10	23	4	**2.8**	**[0.8,10.6]**
26 weeks after start therapy	Kwakkel	Max walk speed	26	0.9 (0.7)	34	0.6 (0.6)	0.3	[-0.0,0.6]
**Follow-up 2**								
6 months after start therapy	Lincoln	ARAT	81	3.0 (28.9)	81	19.0 (33.3)	**-16.0**	**[-25.6,-6.4]**
52 weeks after start therapy	Kwakkel	ARAT	28	6.0 (31.3)	33	1.0 (21.1)	5.00	[-8.6,18.7]
6 months after stroke	Rodgers	ARAT	48	55.0 (31.9)	48	56.0 (23.7)	-1.0	[-12.2,10.2]
	***Subtotal - ARAT***		157		162		-6.4	[-12.8,0.0]
6 months after start therapy	Lincoln	10 Hole Peg Test	81	0 (40.7)	81	0 (45.2)	0.0	[-13.3,13.3]
6 months after stroke	Rodgers	Frenchay Arm Test	48	5.0 (3.0)	48	4 (3.0)	1.0	[-0.2,2.2]
6 months after start therapy	Lincoln	Rivermead arm	81	4.0 (6.7)	81	6.0 (5.9)	**-2.0**	**[-4.0,-0.1]**
6 months after start therapy	Lincoln	Rivermead Gross Function	81	6.0 (5.9)	81	7.0 (3.7)	-1.0	[-2.5,0.5]
52 weeks after start therapy	Kwakkel	Max walk speed	25	0.9 (0.6)	33	0.7 (0.6)	0.2	[-0.1,0.5]
52 weeks after start therapy	Kwakkel	FAC	25	5 (0.7)	33	4 (1.48)	**1.0**	**[0.4,1.6]**
6 months after start therapy	GAPS	Rivermead mobility	30	10.2 (3.1)	34	9.1 (4.0)	1.1	[-0.6,2.8]
52 weeks after start therapy	Kwakkel	Comfort walk speed	25	0.6 (0.5)	33	0.5 (0.4)	0.1	[-0.1,0.3]

^a ^= fixed effect model used; ^b ^= random effect model used; ^c ^= odds ratio used; FU = Follow-up; * = = < 0.05; ARAT = Action Research Arm Test; FAC = Functional Ambulation Category

At outcome, data from one trial relating to Rivermead Mobility Index was omitted because only 3 of 35 participants in the extra therapy group appear to have been included in the outcome data compared to all participants in the control group [22]. Therefore values provided may not have been representative of the entire group. Meta-analysis was undertaken for Action Research Arm Test (3 studies) and comfortable walking speed (2 studies) with effect sizes of 0.1 (-5.7,6.0] and 0.3 [0.1,0.5] respectively. For other measures, the individual study comparisons found a trend towards a better outcome with higher dose for most comparisons but this was weaker than for motor impairment- muscle function. Significance was only found in individual study comparisons in favour of extra therapy for: ability to walk at 0.8 m/sec or more with an odds ratio of 3.9 [1.1,13.9] and maximal walking speed effect size, 0.4 [0.1,0.7]. A significant benefit for standard dose therapy was found for one individual study comparison for the Rivermead Gross Function score with effect size -2.0 [-3.4,-0.6].

At follow-up-1 meta-analysis was undertaken for Action Research Arm Test (2 studies), Rivermead Mobility Score (2 studies) and comfortable walking speed (2 studies) with non-significant effect sizes of 2.2 [-6.0,10.4], 1.0 [-0.6,2.5] and 0.2 [-0.1,0.4] respectively. For other measures the significant effect sizes from individual studies were: Rivermead Arm score, -2.0 [-3.7,-0.3]; 5 metre walk time, -13.6 [-26.2,-1.0]; Functional Ambulation Categories, 1.0 [0.2,1.8]; and ability to walk at 0.8 m/sec or more, 2.8 [0.8,10.6].

The follow-up-2 meta-analysis (3 studies) found a significant benefit for standard dose therapy for ARAT, subtotal of -6.4 [-12.8,0.00]. A significant benefit in favour of standard dose therapy was also found from an individual study in respect of the Rivermead Arm score with an effect size of -2.00 [-4.0,-0.1]. The benefit for higher dose therapy was, however, maintained for Functional Ambulation Category, 1.0 [0.4,1.6].

## Discussion

This systematic review provides limited support for the hypothesis that a higher dose of exercise-based therapy enhances motor recovery after stroke. There are some indications from the present meta-analysis for benefit from a higher dose for: comfortable walking speed; maximum walking speed; and upper limb muscle function. Meta-analysis was, however, limited by heterogeneity between studies in the measures used and therefore most estimates of effect size were derived from single studies. Those single study sample estimates that were statistically significant were mostly in favour of a higher dose of therapy. In contrast, there are also some indications from meta-analysis for benefit from a standard dose for hand grip force/strength and upper limb functional ability at outcome (Table 6) and for ARAT score at follow-up 2 (Table 8). Caution in interpretation of the results of the present review is also raised by the finding that only three of the seven included studies had all design elements assessed as low risk of bias. Clearly there are limitations to the current evidence base that restrict the provision of clear guidance for whether an increased dose of exercise-based therapy enhances recovery after stroke.

This finding differs from the results of benefit from extra therapy of: earlier systematic reviews [1-5] and experimental human studies (for example [12]). But, maybe this result is not so surprising considering that animal model studies and a clinical trial found that higher doses of therapy produced worse outcomes early after stroke [6-10]. Interestingly the detrimental effect found in the present systematic review on hand grip force/strength (Table 6) and upper limb function as measured by the ARAT (Table 8) emanate from trials conducted early after stroke [21,24]. It is possible therefore that there is a negative interactional effect between time from stroke and dose. However, other trials included in the present review were also conducted with participants early after stroke and detrimental effects were not found for either upper limb or lower limb motor impairment or activity. Prospective robust clinical trials are needed to investigate whether time after stroke influences motor response to different doses of exercise-based therapies.

A starting dose for subsequent trials is suggested by an earlier systematic review which concluded that a 16-hour difference in treatment time between experimental and control groups provided in the first 6 months after stroke is needed to obtain significant differences in activities of daily living" [3]. Investigation of the data reported here for a potential dose-response relationship is limited by the relatively small number of comparisons that can be included in a meta-analysis because of the variation in measures used in included studies. We were concerned to avoid undertaking analyses of sets of heterogeneous measures in a single meta-analysis. However, visual inspection of outcome time-point data (Tables 6,7 and 8) and data on dose (Table 4) suggests a trend for better outcome with higher dose. The highest doses, however, were of task-specific interventions [18-20] whereas the smaller doses consisted of UK conventional physical therapy [25,26]. This difference could have influenced the results of the present review. It is also possible that differences in effect sizes between studies could be due to differences in underlying standard care. The study by Kwakkel and colleagues [18-20] was conducted in the Netherlands whereas the other four studies took place in the United Kingdom. This could have influenced the results of the present review because there may be important differences in underlying routine care between centres and countries [27]. There may also be differences in standard therapy over time [28]. Therefore the differences in clinical setting for studies may also be influential on outcome. Consequently, this present review which restricted included studies to those investigating different doses of the same therapy to avoid the confound of different types of therapy may itself be confounded by the inclusion of different types as well as different intensities of therapy. Essentially this systematic review highlights the need for prospective dose-ranging studies of specific interventions before undertaking efficacy studies.

None of the doses investigated in included studies emerged from preliminary dose-finding studies. The same observation emerged from in a systematic review and meta-analysis of electrostimulation [29]. Indeed dose-finding has not featured prominently as a precursor to stroke rehabilitation trials [30,31] Without precursor dose-finding studies it is possible that the studies included in this review investigated sub-optimal doses of exercise-based therapies. The case for prospective dose-finding studies as precursors to Phase II and phase III trials of rehabilitation has been made already [30,31]. Nevertheless, we are aware of only one study designed to investigate the relative efficacy of three or more doses of the same rehabilitation therapy [Hunter SM, Hammett L, Ball S, Smith N, Anderson C, Clark A, Tallis R, Rudd A, Pomeroy VM. Appropriate dose of Mobilisation and Tactile Stimulation to enhance upper limb recovery early after stroke: a randomised controlled trial. Submitted]. Dose-finding has not featured prominently as a precursor to phase II and phase III trials of rehabilitation therapies.

A potential limitation to the present review is the examination of multiple data sets from the same study participants. Therefore bias is potentially present through the repeated use of results arising from the same group of participants. In recognition of this possibility the present review did not combine data from the same studies within meta-analyses.

Another potential limitation is that the present review may be influenced by a publication bias as the literature search excluded studies written in a language other than English. A strong publication bias is, however, unlikely to be present the studies included in this present review were also included in previous meta-analyses. In addition, authors of included studies were contacted for any unpublished data.

## Conclusions

The findings indicate that there is limited empirical evidence to inform clinical decisions on how much exercise-based therapy is needed to enhance motor recovery after stroke. To the best of our knowledge the present systematic review of the effects of dose of therapy is the first to control for the potential confounder of different types of intervention. It has refined and updated knowledge of the effects on motor recovery of the provision of an increased dose of exercise-based therapy after stroke. It has highlighted the clinical uncertainty around dose. Further systematic reviews are unlikely to resolve this clinical uncertainty because of the heterogeneity between exercise-based therapies in included studies and the apparent lack of dose-finding studies undertaken as precursors to robust clinical trials. The results of the present systematic review therefore indicate a need to undertake dose-finding studies of specific exercise-based interventions as precursors to robust clinical trials.

## Competing interests

We gratefully acknowledge: funding provided by The Healthcare Foundation and St George's Charitable Foundation that enabled us to undertake this study. The authors declare that they have no other competing interests.

## Authors' contributions

Dr Cooke has made substantial contributions to conception and design, acquisition of data, analysis and interpretation of data, revision of manuscript, and has given final approval of the version to be published.

Kathyrn Mares has made substantial contributions to acquisition of data, analysis and interpretation of data, revision of manuscript and has given final approval of the version to be published.

Dr Clark has made substantial contributions to analysis and interpretation of data, revision of manuscript, and has given final approval of the version to be published.

Professor Tallis has made substantial contributions to conception and design, revision of manuscript, and has given final approval of the version to be published.

Professor Pomeroy has made substantial contributions to conception and design, acquisition of data, analysis and interpretation of data, revision of manuscript, and has given final approval of the version to be published.

## Pre-publication history

The pre-publication history for this paper can be accessed here:

http://www.biomedcentral.com/1741-7015/8/60/prepub
